# Community Acceptance of Tsetse Control Baits: A Qualitative Study in Arua District, North West Uganda

**DOI:** 10.1371/journal.pntd.0002579

**Published:** 2013-12-12

**Authors:** Vanja Kovacic, Inaki Tirados, Johan Esterhuizen, Clement T. N. Mangwiro, Stephen J. Torr, Michael J. Lehane, Helen Smith

**Affiliations:** 1 Department of Vector Biology, Liverpool School of Tropical Medicine, Liverpool, United Kingdom; 2 Department of Animal Science, Bindura University of Science Education, Bindura, Zimbabwe; 3 Warwick Medical School, University of Warwick, Coventry, United Kingdom; 4 Department of International Public Health, Liverpool School of Tropical Medicine, Liverpool, United Kingdom; University of Pittsburgh, United States of America

## Abstract

**Background:**

There is renewed vigour in efforts to eliminate neglected tropical diseases including sleeping sickness (human African trypanosomiasis or HAT), including attempts to develop more cost-effective methods of tsetse control. In the West Nile region of Uganda, newly designed insecticide-treated targets are being deployed over an area of ∼500 km^2^. The operational area covers villages where tsetse control has not been conducted previously. The effectiveness of the targets will depend, in part, on their acceptance by the local community.

**Methodology/Principal Findings:**

We assessed knowledge, perceptions and acceptance of tsetse baits (traps, targets) in villages where they had or had not been used previously. We conducted sixteen focus group discussions with male and female participants in eight villages across Arua District. Discussions were audio recorded, translated and transcribed. We used thematic analysis to compare the views of both groups and identify salient themes.

**Conclusions/Significance:**

Despite the villages being less than 10 km apart, community members perceived deployed baits very differently. Villagers who had never seen traps before expressed fear, anxiety and panic when they first encountered them. This was related to associations with witchcraft and “ghosts from the river” which are traditionally linked with physical or mental illness, death and misfortune. By contrast, villagers living in areas where traps had been used previously had positive attitudes towards them and were fully aware of their purpose and benefits. The latter group reported that they had similar negative perceptions when tsetse control interventions first started a decade ago. Our results suggest that despite their proximity, acceptance of traps varies markedly between villages and this is related to the duration of experience with tsetse control programs. The success of community-based interventions against tsetse will therefore depend on early engagements with communities and carefully designed sensitization campaigns that reach all communities, especially those living in areas new to such interventions.

## Author Summary

Sleeping sickness is a disease which results in serious physical and mental symptoms and is ultimately deadly if not treated. It is caused by sub-species of *Trypanosoma brucei* transmitted by tsetse which live exclusively in Africa. Currently, the only preventive measure against sleeping sickness is reduction of tsetse population in the areas where these flies and humans share the same living space. This can be achieved through the use of traps or insecticide-treated targets to attract and kill tsetse. As the traps are newly introduced in some areas, we explored how local communities perceive them. We compared their views to those of communities living in areas where traps have been used sporadically for more than 10 years. Despite villages with or without experience of vector control being less than 10 km apart, they had very different perceptions: the group new to targets had many negative perceptions, associated with witchcraft and supernatural powers, while the group knowing targets from the past perceived them positively and beneficial. Understanding of local perceptions is important, because it will help us to involve communities affected by sleeping sickness in tsetse control programs. Without their support these programs are short-lived and ineffective.

## Introduction

Sleeping sickness (Human African trypanosomiasis or HAT) is a disease that is restricted to the African continent. The disease is caused by sub-species of *Trypanosoma brucei* transmitted by tsetse flies (*Glossina*). *T. b. gambiense* causes a chronic form of sleeping sickness found in West and Central Africa, including the area where this study was conducted. Tsetse are also vectors of other species of *Trypanosoma* pathogenic to livestock and estimated to causes economic losses of US$4.5bn per year [1]. Individuals with HAT, experience a range of physical and mental symptoms, which result in death if not treated. Because of these negative health impacts, HAT is ranked high in terms of burden of disease expressed as disability-adjusted life years (DALYs) [2]. In addition to DALYS, the disease can also have substantial socioeconomic impact on households. Despite the existence of effective vector control measures, HAT remains endemic in 36 countries across Africa [3].

In the absence of prophylactic vaccines or drugs, the only means of preventing infection is to control the vector. Several proven methods of tsetse control exist; for local communities in HAT-endemic areas, the most commonly used and feasible methods are the use of baits (insecticide-treated livestock, traps and targets) to attract and kill tsetse. Many of the foci for Gambian HAT are in areas where livestock densities are low and hence the only method available is the use of traps and targets.

Recent research [4]–[6] has resulted in new designs of target which offer the prospect of more cost-effective means of reducing densities of tsetse and hence risk of HAT. Combined with the renewed global interest in elimination of sleeping sickness by 2020 [7], [8] tsetse control is emerging as an important component in these efforts [9], [10]. As tsetse control technology is becoming more cost-effective, and easier to deploy and maintain under field conditions [11], this makes it an attractive option for large-scale HAT control operations. The current study is a baseline assessment and is part of a bigger trial, evaluating the cost-effectiveness and sustainability of a tsetse control operation over ∼500 square kilometres in north-west Uganda and Boffa focus in Guinea using small and more cost-effective tsetse targets.

Drugs and other disease control measures like insecticide treated nets for malaria, are often considered ‘public goods’ [12] in that the benefits of implementing these measures extend beyond individuals to entire communities and geographic areas. In large-scale vector control operations, decreases in the density of tsetse are likely to reduce risk of HAT benefiting all individuals living within that area. In many countries the potential benefits of this public good are not fully realized. This may be due not only to the lack of sustained government funding for coordinated and large-scale field operations, but also community behaviour or reaction to the technology. For example, communities may remove, destroy, or vandalise targets for a number of reasons, but most often because they have not been adequately consulted, sensitised, or involved in the deployment of the technology. A first step in resolving this is to understand community perceptions of the control tools and use this to enhance community understanding and participation in vector control programmes.

The role of community participation in the success of such large vector-control programs is evident and has its origin in the WHO Alma Ata declaration in 1978 [13], [14]. In the same declaration, the need for cultural appropriateness of health-delivery programs was emphasized as essential for community participation to occur. Similarly the concept of community as partners rather than passive beneficiaries has been promoted in control of vector-borne diseases [14]. The principle of community participation assumes shared responsibility of disease control through access to information, resources and decision making power which are handed over to the target communities [15]. In practice however, collaboration between programmatic and community approaches are still scarce, which is manifested in clashes of expectations, differences in perceived priorities and disparities between local and external definitions reported from different disease control programs (see for example [15]–[19] ). This highlights the need for reinforced communication and mutual collaboration between provider and beneficiary, or rather a need for blurring the boundaries between them. One way to achieve this is to provide space for local communities to express how they perceive and understand objectives of health interventions and the tools used to achieve these objectives.

There are several examples of efforts to enhance community participation in tsetse control baits ranging from encouraging people not to temper with baits through to more active participation in terms of construction, deployment and maintenance of traps (Uganda [19], Kenya [20], Democratic Republic of Congo [21], Ivory Coast [22], Ethiopia [23] and Sudan [24]). In all these studies, community acceptance of traps was a crucial element for the success of these interventions. In contrast, an example from DRC showed that negative attitudes of villagers towards traps, and association with indigenous beliefs, led to damage, theft and vandalism of traps and ultimately operational failure [25]–[27]. Hence, community acceptance of traps is a vital element for effective tsetse control.

We undertook a study in an area of West Nile located in North-West Uganda, where traps have been used in government-supported programs against HAT. We explored acceptance of traps by the local communities in order to identify: i) factors that are likely to enhance acceptance of traps and ii) probability of change in attitude if acceptance of baits by the community is low. With the prospect of community involvement in tsetse control operations, and the potential of such interventions being extended to the new geographical areas, we develop recommendations for policy makers and practitioners in planning and implementing tsetse control programs. We expect that these recommendations will increase the probability of sustainable and effective HAT control operations in the future. The lessons learned for control of tsetse could be applied to other vector-borne and neglected tropical diseases.

## Methods

### Ethics approval

The study protocol and procedures for obtaining participants' consents were approved by the Research Ethics Committee of Liverpool School of Tropical Medicine (ref: 11.73) and Uganda National Council of Science and Technology (UNCST) Ethics Committee (ref: SS-2561). Local district and sub-county administrative authorities and village chiefs were informed about the study and their permission sought prior to data collection. All participants were informed about the study, and encouraged to ask questions; their voluntary participation and right to withdraw from the study were emphasised and their written consent was obtained. In case of illiterate participants a fingerprint was collected in front of a literate witness. Signed or finger-printed consent forms are stored securely at the offices of the LSTM tsetse research project, Arua, Uganda.

### Study area

**Figure 1:**
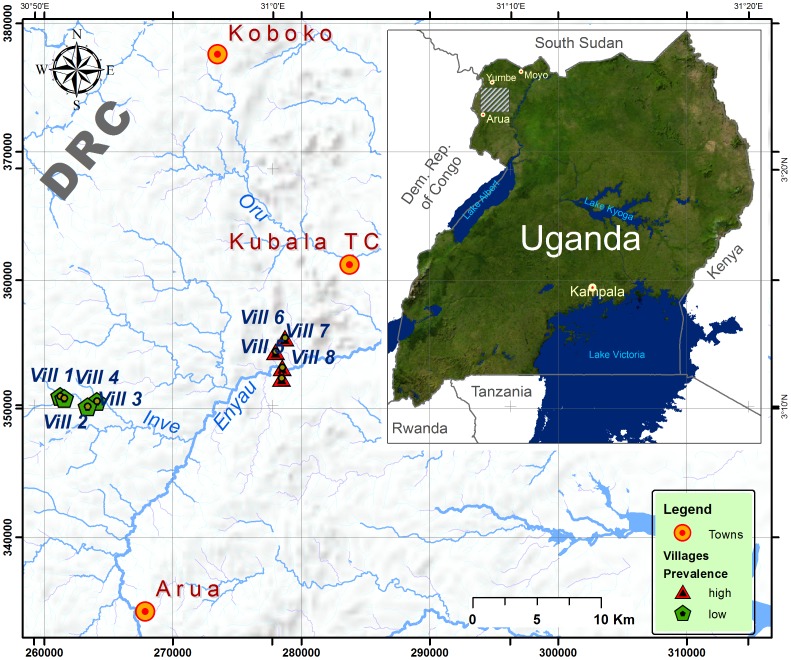
Map of the villages included in the study. A group of the villages with the history of sleeping sickness is indicated by red triangles and the group without history of HAT in green pentagon. Note that both groups of villages lie in proximity of 10

A qualitative study using focus group discussions (FGDs) was carried out in Arua District (03°10′N-30°52′E-03°12′N-31°00′E) in West Nile, Uganda (Figure 1). The study site was purposively selected because it is a known historical and currently active focus for gambiense sleeping sickness [28]. The Lugbara are the predominant ethnic group in the area, with the minority Kakwa group located in the northern part of West Nile, close to the South Sudan border. The population is engaged primarily in mixed crop-livestock farming; the main subsistence crops are cassava, maize, beans and sweet potatoes. Tobacco is grown as a cash crop and planted in some areas. Goats and cattle breeding are common throughout Arua District with some pig breeding in non-Islamic villages. Small scale fishing in the local rivers and streams is carried out for domestic consumption rather than for commercial purposes. According to population projections for 2013, Arua District has a population of about 801,400 people (unpublished Arua District Report). The main religious orientations are Christian (Catholic, Anglican Protestant) and Muslim.

We selected two groups of villages in Arua district based on availability of data on historical and current HAT transmission and their exposure to tsetse control programs. One group comprised four villages in HAT affected areas. In these villages, in 2010 (i.e. within 12 months of the study) Médecins sans Frontières (MSF) carried out a targeted screening and treatment program. These villages are coded HAT+ for the purpose of this study and MSF detected an average prevalence of 0.5% respectively (prevalence between 0.3% and 0.8% with the active screening coverage between 63% and 89%). The other group comprised villages with no recent or historical cases since 2000 (MSF, unpublished report; Omugo Treatment Centre medical records). Hence, this area is considered to have little-active HAT transmission and was therefore excluded from MSF active screening plan. In our study we coded these villages as HAT−. Table 1 shows the characteristics of the selected villages including HAT prevalence threshold.

**Table 1:** Characteristics of the study villages and data collection method.

Village code	Population*	Detected HAT prevalence in 2010**	Village group	FGD Number	Participants' gender	Nu. of participants
1	262	/	HAT−	1	F	9
				2	M	9
2	473	/	HAT−	3	F	12
				4	M	8
3	359	/	HAT−	5	F	10
				6	M	9
4	363	/	HAT−	13	F	10
				14	M	8
5	399	0.3 or above (63–89% active screening coverage)	HAT+	7	F	10
				8	M	9
6	834	0.3 or above (63–89% active screening coverage)	HAT+	9	F	10
				10	M	9
7	392	0.3 or above (63–89% active screening coverage)	HAT+	11	F	12
				12	M	9
8	570	0.3 or above (63–89% active screening coverage)	HAT+	15	F	12
				16	M	9

This table distinguishes two groups of the villages: those located in the current HAT foci and were in contact with tsetse control programs (HAT+) and those not located in the current HAT foci and without exposure to previous tsetse control programs (HAT−). In addition gender and number of participants per FGDs per village is illustrated.

* Source: Sub-county's Demographic Databases (unpublished).

** MSF Spain Report (unpublished).

Since tsetse control operations are not localized or specific to HAT endemic villages, we considered another selection criterion. The other major difference between the two groups was exposure to tsetse control operations, which had been carried out intermittently in the HAT+ area over the last decade by NGOs (MSF France in the 1990s) and recently by local government agencies. About three years before this study 70 traps on average per year were deployed per parish (about 10 traps in villages along the river) (personal communication with Arua District Entomologist). However, tsetse traps were not regularly monitored and maintained, hence theses efforts did not result in sustainable tsetse control in this area. Trap deployment, however was frequent enough to enable communities in HAT+ villages to familiarize themselves with traps. In HAT− villages no previous tsetse control programs have ever been carried out, therefore, the first exposure to tsetse control baits was introduced as a part of this study.

In total, eight villages were included in the study, four from the HAT+ area and four from the HAT− area. Location of the villages and the distance between them is shown in Figure 1. In HAT− villages two pyramidal traps per village were deployed in the central point of the villages 100 meters apart at the bank of the river running through the villages. These traps were deployed about three weeks before the study was conducted and remained on the sites for the whole pre-study period. This was carried out in order to expose villagers to traps before beginning of discussions. No previous sensitization activities had been carried out prior to this deployment or data collection. In HAT+ villages no new traps were deployed specifically for this study, but we observed about six traps along the river running through our study villages, which remained deployed from the previous control operations mentioned above.

### Data collection

Sixteen focus group discussions (FGDs) were carried out in December 2011. In each FGD we explored whether communities considered HAT a public health problem, if they were aware of the role of tsetse in transmission, and how they perceived tsetse traps. A topic guide was used and pretested in two informal FGDs, followed by refining the topics. Final topics explored included:

- Local knowledge, attitudes and practices associated with traps and HAT control interventions.
- Acceptance of traps and potential change of acceptance over time.
- Perceived benefits of traps and willingness to be involved in tsetse control activities.

In order to capture gender-specific views and encourage participants to talk openly and freely, we conducted separate FGDs with males and females in each village. Table 1 shows the characteristics of the villages as well as composition of the FGDs.

**Figure 2:**
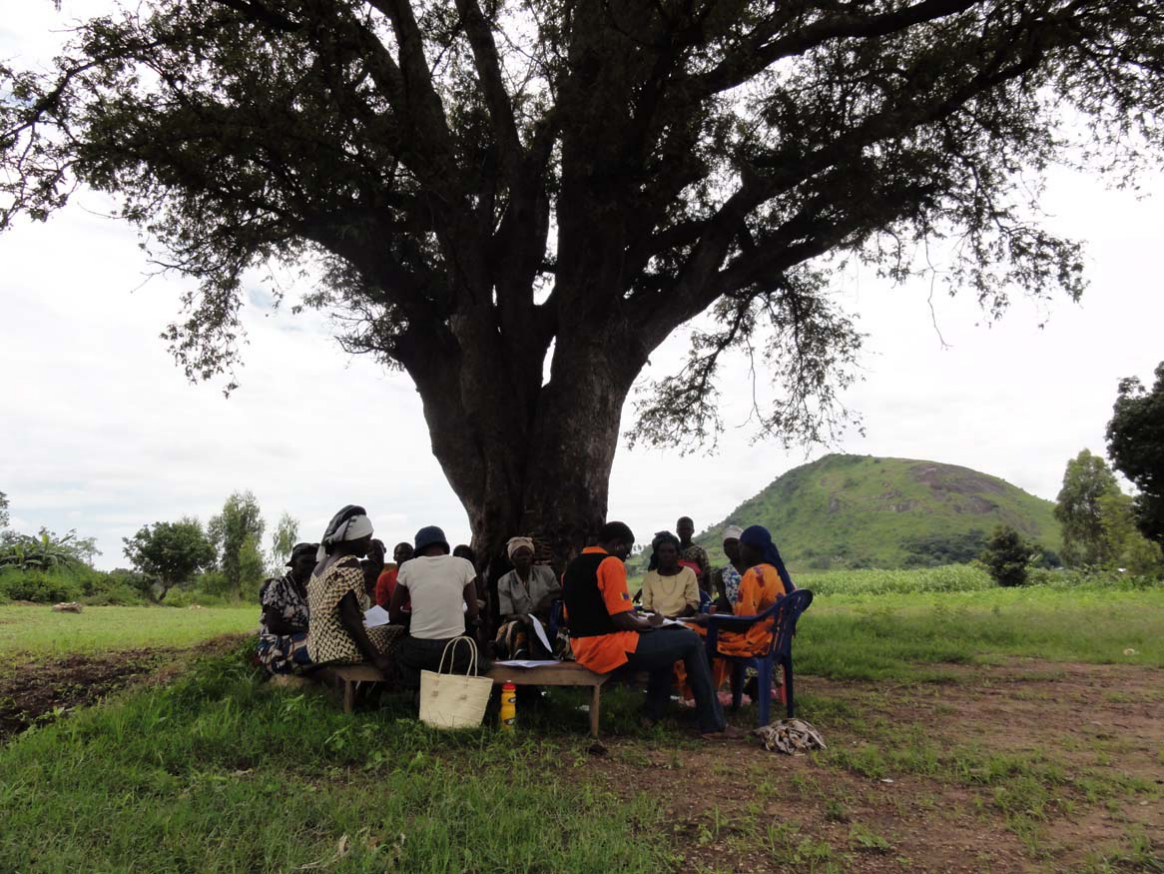
Picture of the female focus group discussion (FGD) in one of the study villages. FGDs were run in the usual village meeting place under the tree.

On average, ten participants per FGD were recruited by the village chief based on gender, age (older than 18), and belonging to different households within the same village. FGDs were carried out at a site where village meetings were usually conducted (Figure 2); the site was identified by the village chief.

Discussions lasted about an hour and were conducted in Lugbara and simultaneously translated into English by a trained translator. All FGDs were led by the main researcher (VK), and two field assistants: a facilitator/translator and observer/note taker. Facilitator and observer are both fluent in English and Lugbara and trained in social science research methods. All discussions were audio recorded by digital voice recorder, with permission of the participants.

### Data analysis

Audio recordings were transcribed into Word documents by the field assistants. In the process of transcription all the audio translations were cross-checked and supervised by VK to ensure the accuracy of the final transcripts. At the beginning of the analysis process, VK read all transcripts at least twice to identify main themes and/or and until no new themes were identified; VK and HS discussed and refined the list of themes. In the next step of the analysis, VK used the list of themes to code all of the transcripts; this process was managed using MAXQDA 10 Software [29]. After the coding process was completed, we organised the coded segments of data into tables (thematic matrices), using one table per theme. VK examined each thematic matrix to identify patterns in the data; for example, any differences or similarities in views between men and women or between the different villages. VK then described each theme in detail and identified typical quotes to illustrate the meaning of the themes. In the results section we present the five main themes with illustrative quotes.

## Results

Villages from HAT+ and HAT− areas lie within 10 kilometres of each other. Villagers from different villages often mix in the local markets and during social events such as celebrations of country independence, women's day, Christmas; hence the differences in views described below were surprising.

### i) Knowledge and perception of HAT

#### Perception of HAT as an important problem in the community

A vast majority of participants from HAT+ villages reported HAT to be among the three leading diseases in their community, following malaria and typhoid. On average, respondents reported witnessing over forty HAT cases per village over a period of their life-time. In addition many of them had a personal experience looking after HAT patients in their families. The high number of cases was reported mostly from the 1990s, however in the recent years, villagers reported a decline in disease incidence.

By contrast, villagers in the HAT− area mostly regarded HAT as disease of the “old days” and reported never having witnessed it. The only reference to HAT were stories from their parents, who talked about “neck examination” (examination of lymph nodes) organized by the colonial authorities in this area in the past. Participants therefore mostly concluded that HAT is not a major public health problem in their area.

Participants in both groups used the following criteria for measuring the importance of a disease in their communities: high rate of infection, fast developing disease with potential to kill, and personal experience with disease, especially witnessing a sick child.

#### Knowledge of HAT symptoms and epidemiology

The villagers differed in their knowledge of the transmission of HAT. In HAT+ areas, all participants agreed that HAT is transmitted through tsetse and were able to describe the transmission process. In addition they demonstrated a detailed knowledge of HAT symptoms; mental disturbance, headaches, and swellings of the body were the most commonly mentioned.

In contrast, knowledge on HAT symptoms and transmission was lacking among villagers in HAT− areas. The majority were not able to mention any HAT symptoms, with a few noting that extensive sleep is one of them. Similarly the role of tsetse in transmission of HAT was unclear to them. Participants reported other routes of transmission such as through mosquitoes, food, animal blood from an insect bite, water, rats and by drinking strong alcohol. One participant assumed that tsetse transmit malaria and HIV.

Participants reported that information about HAT was mostly spread through active screening sensitization campaigns in HAT+ areas, while in HAT− areas participants had acquired some information from their parents or in school. There did not appear to be any difference between knowledge of HAT between men and women from either group.

### ii) Perceptions of community co-existence with tsetse

In both groups, all participants reported being aware of tsetse flies. The majority of participants described two or three different groups of biting insect, which they classify under category of tsetse (Lugbara, wii or ofii). The subcategories included “the infectious tsetse” and non-“infectious tsetse”, the last being mostly recognized as causing biting nuisance to humans and cattle. The “infectious tsetse” were mostly described as big in size, having sharp mouth parts, lighter in colour compared to other flies, by participants of both groups.

After the initial discussion a sample of dry tsetse flies in petri-dish was shown to participants to probe further discussions. Based on this, both groups recognized tsetse preference for dark areas, bushy and forested vegetation, with valleys, river banks and other water bodies being reported as the most frequent habitats. Some participants noticed that tsetse move around with humans and cattle and described this route as being responsible for introducing tsetse near their homes. Only one participant from the HAT− group recognised cattle as a potential host for tsetse.

Participants from both groups reported that being attacked by tsetse occurs when people enter a habitat with tsetse. One participant, for instance, mentioned that other flies would be attracted to wounds, while tsetse flies approach humans regardless of any other stimuli apart from human presence. Participants from both groups did not reach consensus on tsetse peak biting times.

Participants also described the biting experience of tsetse different compared to experience with other biting insects. Both groups noticed that the bite was quick and, unlike other insects, the bite occurred only once and without the fly returning to the same host. Both groups mostly reported the bite as being painful, involving bleeding, and commonly produced an allergic reaction, such as localized swelling and itchiness. Both groups agreed that naked parts of human body are mostly prone to biting, and some HAT− participants commented that biting also occurs through clothes. The most commonly bitten parts of the human body reported were: back and legs (HAT+) and arms (HAT−); the head was also mentioned as one of the preferred biting spots, by one participant.

In villages within the HAT− area, other associations included tsetse as being strong, dangerous (with the potential to kill), horrifying, and that they bite by surprise. In addition, some participants from both groups also expressed feeling fed up with co-existing with them.

### iii) Fear, suspicion and associations with supernatural powers

Traps deployed along the rivers in HAT− area about three weeks before the focus group discussions - were quickly noticed by the community. Participants reported that seeing unknown blue and black objects caused strong reactions locally, with people expressing feelings of fear, anxiety or panic. Some reported being suspicious, surprised and feeling unease. A few participants expressed curiosity and uncertainty about them. One participant expressed feeling angry and provoked when seeing a trap near his house and one remained worried. Women mostly expressed curiosity and hence approached traps more often, while men mostly expressed feeling fear and anxiety. The quotes below illustrate how villagers initially perceived the traps:

*“Everyone was afraid [when seeing traps], not only one person”* (male, FGD2, HAT−).

*“If you don't know [an object] you can get scared. So we feared, of course”* (female, FGD 5, HAT−)!

*“I saw it [a trap] when I went to wash there with another woman and I told the woman they have stacked something there (…); and she first ran from here up to there”* [pointing to the distant direction] (female, FGD3, HAT−).

The suspicions and fear were not reported to be in any way related to the appearance of traps, but rather its position next to the river, uncertainty about the person who carried out the deployment and the unknown purpose of this action, as one of the participants explained:

*“Since it's just a cloth, there would not be much fear… The fear would be: who has placed it there! For what [purpose]”* (male, FGD 4, HAT−)?

*“As for me, before even it [the trap] was hung here, I was going to town and I got one at Enyau river. We all stood there to inquire why this thing was hanging there, what this person [who deployed it] wants to do to us… I was even worried”*(male, FGD 2, HAT−).

Participants of HAT+ group said similar reactions were reported from the early times of trap intervention in their area about a decade ago:

*“Yes, we were afraid; when we saw the traps for the first time”* (female, FGD15, HAT+).

However, despite describing these initial feelings of fear, surprise and curiosity, none of the participants of HAT+ group expressed having negative feelings about traps any more. On the contrary, all participants expressed positive attitudes towards traps, which indicates that attitudes are prone to change over time.

#### Associations of traps with witchcraft and ghosts

Strong emotional reactions expressed by both groups during the first contact with traps were related to the associations of traps with supernatural powers. Participants explained, for instance, that unusual objects, activities or “suspicious” people moving in their environment made them apprehensive. According to participants of both groups, these objects, activities and people could be associated with two forms of supernatural powers: i) witchcraft (Lugbara: “ojo azi ngazu”) and ii) ghosts from the river (Lugbara: “*orindi onzi*”; *orindi*-ghost/spirit, *onzi*-evil/bad).

Associations with witchcraft were only mentioned by the HAT− group and by men more often than women. There were other elements which reinforced the idea of witchcraft, such as a goat recently being killed near the position of the trap and thrown into the river. This was interpreted as a type of sacrifice commonly used by people “who practice witchcraft”. Traps being deployed in the area which is locally known for its witchcraft practices also increased the suspicion, for example:

*“The first time [I saw the trap] I thought of witchcraft because there is witchcraft in this area; so I thought* t*hese people have come to practice witchcraft here”* (female, FGD 5, HAT−).

*“The reason we feared was, since this is a bad season or period, there are these people of witchcraft in* the *form of magi [which doctor], who came to fix these things… That's why we feared so much”* (male, FGD 2, HAT−).

Members of the HAT+ group similarly reported initial associations with ghosts, but only related to the first period of deployment. These associations were reported as frequently as the HAT− group. Position of traps next to the rivers was critical for these associations since the river was described as being the home of ghosts. Ghosts were described as manifest in form of light, music, rhythms of dance, or in form of a human being who is lighter by skin colour and hairier than normal. The quote below is typical of the views shared in the FGDs in both areas:

*“The issue of ghost comes in those [parts] of the days when it gets dark, or like it's late in the evening; and you are passing the river, and you could find light either inside the river, or along the river side, but you could not know the source of light. And then some times when passing [there] you could only hear some people talking in the river, without seeing them physically. Sometimes you could hear them singing, and dancing traditional songs or any other music, yet they could not be seen! (…) In the early times, they used to slash the areas along the river to keep it clean, but now if you find this trap-the cloth-colours blue and black hanging at the river side, this strange system will scare you wondering what it is, who hung it there, and for what purpose? You may easily associate this to the ghosts since the strange voices have been heard talking singing, and noise and rhythms of dancing all from the river. This is what scares the people who meet the traps for the first time before being told what it is”* (female, FGD 9, HAT+).

Coping mechanisms for these first strong emotional reactions were described by both groups. Participants would for instance report running away from the site of the traps, refusing to fetch water, avoiding the area of traps, praying in the name of Jesus and trying to gather information from the neighbouring homes.

### iv) Community perceptions of traps and risk

To understand these reactions further, we explored perceptions of risk in both communities. Villagers in HAT− areas perceived risk to their wellbeing when in close proximity of traps. This was not directly due to the presence of traps or their appearance, but due to traps being associated with supernatural powers as explored above. According to the participants, performance of witchcraft and contact with evil ghosts poses a major risk for human health, causing physical or mental illness, death, and misfortune:

*“When you meet such bad luck at the river (ghost or witchcraft) you either die or fall sick”* (male, FGD 14, HAT−).

*“Yeah, ghosts are dangerous. Sometimes they can beat you, or make you dumb [mad] so that you may fail to speak to people for two to three days”* (female, FGD 9, HAT+).

The presence of white foreigners used to be perceived risky as well, as one of the participant from HAT+ village described:

*“I was thinking out of fear; whites have brought these things maybe to kill us… I didn't know in which way the killing would happen”* (female, FGD 11, HAT+).

The idea of not being allowed to touch traps was also commonly reported. While in the HAT− group touching a trap was perceived as a risk of dying in a very abstract way, in HAT+ group physical contact with the trap was always associated with the risk of insecticide affecting ones skin or punishment from the local authoritative organs in terms of imprisonment as quotes below demonstrate:

*“Then they said if you touched this thing you will die; so everyone was running away from it”* (female, FGD 3, HAT−).

*“I thought it was sprayed with vaccine [insecticide] and once you touch the vaccine [insecticide] it will have effect on you which could be negative. (…). I was told also if you touched the trap you will go to prison”* (female, FGD 15, HAT+).

#### Perception of traps' purpose, function and effectiveness

In the group discussions we used a sample trap to prompt discussion on trap function and purpose. A small minority of participants could not offer any explanation on the function of traps, but among those there was no difference between HAT+ and HAT− group or between men and women. Some participants however, knew or made correct assumptions on how flies get captured in the upper cage flying towards light. Blue and black colours were correctly described as attractants by many of the participants from both groups.

The majority of participants described the purpose of traps for capturing tsetse flies or other insects. In the HAT− group this conclusion was arrived at mostly from observation of trapped insects on in the upper part of the trap. In this group, participants mostly made associations of traps with mosquitoes however some were certain that traps were specifically for killing tsetse flies.

In both groups, the majority of participants reported or assumed that traps work effectively, however the HAT+ group provided more specific details. Participants, for instance, used the following observations for describing effectiveness: reduction in tsetse numbers, reduction of tsetse bites, protection of community against tsetse, and reduction of HAT cases in the area. Participants were also quite specific on suggestions of improved efficacy, such as traps placed with sufficient density and in the right places; bushy areas or next to the households were mentioned as an example.

#### How do community perceptions change over time?

Despite many negative first reactions and attitudes towards traps, participants from both groups expressed that these attitudes are prone to change. Some participants from the HAT− group, for instance reported that, on some occasion their first contact with traps was accompanied by the explanation of the trap purpose or general explanation that traps are beneficial. This communication mostly happened through personnel carrying out trap deployment or another member of community, who had been informed about it previously. According to participants this information helped them to put aside associations with supernatural powers. Among these participants none of them reported experiencing fear, suspicion or worry:

*“I didn't fear! For me, we were interested to be helped; when they [people deploying traps]) said such a thing: (like) they were here to help us, as a community, we were all out to receive that help”* (male, FGD 14, HAT−).

Positive association of deployment-personnel with health workers prompted to similar reactions:

*“I was told it is health workers. People who came around [to deploy targets], so my thought was that they are trying to prevent any disease that is coming from the rivers”* (male, FGD 14, HAT−).

Being able to observe insects being trapped, which was mostly reported by female participants, also seemed to influence positive attitudes:

*“[When I saw a trap] a lot of insects were gathered there including tsetse flies; but now that thing [idea of witchcraft] went out of my head; I just knew that it is for tsetse flies and it was inserted by the right people [not those of witchcraft]”* (female, FGD 5, HAT−).

Similarly participants from HAT+ villages, communicated changed negative- initial attitudes:

*“Before we heard of them [traps] we didn't like them; but now we have got to know them [the traps], we like them (…). We like them because since that time they deployed them this has reduced the number of tsetse flies in the area”* (female, FGD11, HAT+).

### v) Willingness to be involved in tsetse control

Regardless of the group, participants expressed willingness and appeared motivated to be involved in tsetse control programs; although the HAT+ group expressed this willingness more frequently. Participants mostly requested having access to traps and being involved in their deployment and distribution. The HAT+ group reported that they were already contributing to tsetse control by voluntarily maintaining traps, slashing vegetation around watering points, and burning bushes. More women than men reported being involved in these activities. In both groups women expressed more need for empowerment (see a quote below) and requested access to traps more often, while men expressed more need for village decision making, financial motivation and government involvement in tsetse control programs.

*“Yes… We can participate doing the tsetse control work (here). For example here (in our village), we have a village health team (volunteers)… So if enough traps were given to us, the village health team would look for more, let's say six people to add to the team of two (already recruited as village health team), so that we become eight, and we would unite ourselves to do the work with the team; to do tsetse control intervention”* (female, FGD 9, HAT+).

Some participants in both groups, but more so in the HAT− group, expressed a need for access to HAT testing and drugs.

**Figure 3:**
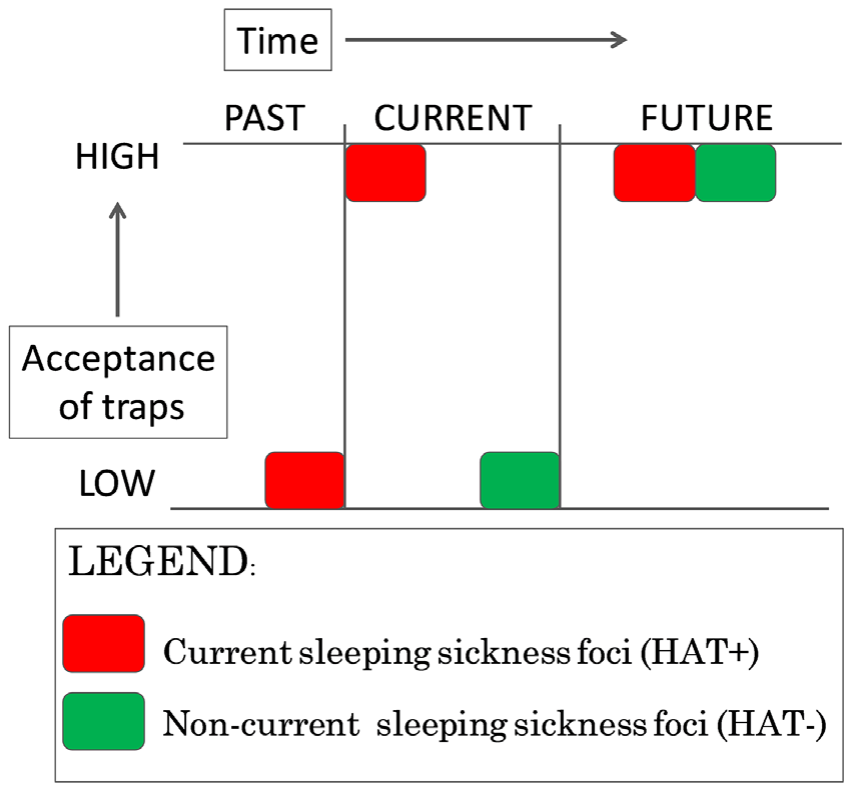
Acceptance of traps in current (HAT+) and non-current (HAT−) sleeping sickness foci. This figure indicates that initial acceptance of tsetse traps when they are initiated in the community is low. This was the case HAT+ villages at the beginning of the tsetse control intervention about ten years ago and is currently still evident in HAT− villages. With the time this acceptance increases and is irreversible once achieved.

Figure 3 illustrates acceptance of traps by HAT+ and HAT− group. The time dimension is plotted with past, current and potential future level of acceptance. Potential future has been evaluated through perceptions of the HAT+ group, in which once high acceptance of traps has been achieved in the past it remained stable over time.

**Figure 4:**
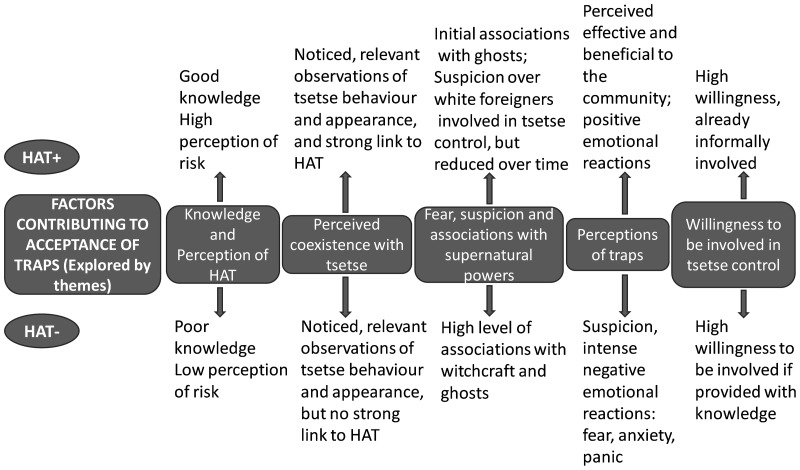
Factors contributing to acceptance of tsetse traps explored by themes. This figure illustrates the five themes identified by the analysis: i) knowledge and perception of HAT, ii)Perceived coexistence with tsetse, iii) Fear, suspicion and associations with supernatural powers, iv) Perceptions of traps and v) Willingness to be involved in tsetse control. Differences between HAT+ and HAT− villages are pointed up. The figure compares how the themes were manifest in current (HAT+) and non-current (HAT−) villages.

Factors contributing to acceptance of traps, explored through emerging themes are shown in Figure 4. Views are compared between HAT+ and HAT− groups and their differences are emphasized.

## Discussion

Despite the near proximity and common interaction of the villagers during trading and celebrations, we found a remarkable difference in perceptions of traps by different villages described below. Villagers newly introduced to tsetse traps perceived them negatively, while only ten kilometres apart communities previously exposed to tsetse control programs unquestionably reported high acceptance of traps. The last group reported that positive acceptability of traps occurred gradually and after initial suspicion and low acceptability by the communities. Therefore it seems likely that negative perceptions are prone to change over time.

Local acceptability of control tools is crucial for the sustainability of control programs. Therefore awareness of socio-economic barriers will have a significant impact on success of such interventions [10]. The findings of this study show that despite the geographical proximity between each other and to the nearest town, two communities belonging to the same ethnic group, with similar occupational activities and level of education differ markedly in their knowledge, attitudes and perceptions of HAT and tsetse trapping technology. The community, who were recently initiated into the tsetse control program, reported significant gaps in knowledge of HAT symptoms and transmission and available HAT control techniques. According to previous studies [30], [31] this knowledge is a crucial determinant of a community's willingness to contribute money or labour for tsetse control programs.

Furthermore, villagers not familiar with traps experienced them as threatening due to the associations with witchcraft and ghosts. This resulted in poor awareness of the purpose of traps and suspicions about the personnel deploying them. Similar findings have been reported from qualitative research carried out in the Democratic Republic of Congo (DRC), where tsetse traps were perceived as untouchable objects associated with supernatural powers [32]. The authors noted that community members were not willing to touch traps claiming that they were objects attracting misfortune and evil supernatural powers. This resulted in villagers refusing to lay traps and traps often being abandoned due to lack of maintenance. These negative associations are not surprising considering that water bodies are often associated with supernatural powers across Africa (see for example: [33], [34]). Unusual looking tsetse baits deployed next to river banks therefore often fuel this connotation. Other extraordinary and coinciding events may also fuel suspicion. In DRC for instance, tsetse trapping coincided with epidemics of swine fever and caused villagers to conclude that the smell of traps caused high mortality in pigs [35].

In contrast, our study shows that in communities where traps have been present for a decade people knew about their purpose and perceived them as being effective and beneficial. In this case no associations with supernatural powers were recorded any more, despite the fact that these negative attitudes were as frequent as in the unexposed group in the initial stage of trap deployment. This difference in the level of awareness could be attributed to previous experience with HAT outbreaks and HAT control programs. This indicates that sensitisation campaigns organized in the past were strictly localised. Surprisingly, despite the high local mobility of the village inhabitants', relevant information has not spread easily to neighbouring villages. A key finding of our study is that negative perceptions and attitudes related to tsetse traps are prone to change; however this change only occurs locally. After we had completed this study, we conducted intense sensitisation (informed by the findings of our study) throughout the research areas.

Our findings have several implications for HAT control policy makers as well as researchers and programme managers carrying out tsetse control activities. Tsetse control activities have so far been carried out by a small group of trained personnel, but more long term strategies, involving local communities and promoting collective action are needed. According to our findings, community members are willing and motivated to contribute towards tsetse control and they requested tools to empower them in these efforts. Motivating factors mentioned by communities involved in this study are in line with those documented by other vector control studies and include: perceived reduction of nuisance biting, reduction of observed number of insects, reduction in cases of disease [36], [37] and perceived effectiveness of technology used [38]. A sense of group cohesion [39] and enthusiasm were identified as additional important elements. Furthermore a study on community participation in efforts against animal trypanosomiasis in Gambia suggested that farmers are more community-oriented than individualistic, when expressing preferences for disease control scheme [40]. A community-centred and culturally sensitive approach in tsetse control has been shown to contribute significantly to the reduction of tsetse flies and incidence of HAT in previous research [19]–[21], [24], [41], [42] and our findings underline the importance of understanding anthropological factors if the full benefits of vector control are to be realized.

Towards this end, our data indicate that tsetse control strategies should address specific cultural requirements. Information needs to be transparent, and focus on: explanation of the purpose of traps and targets, details and reasons for their deployment next to the rivers and demonstrations of tsetse being caught in the traps. Introducing the project field staff would also contribute to building up trust. Sensitisation activities, especially when tsetse baits are used for research purposes, are often absent or coincide with tsetse trap deployment; however results of this study suggest that appearance of the “new object” creates distressing emotions, suspicion and potential tensions within the community; these negative perceptions are long lived and negatively affect not only tsetse control programs but also wellbeing of the host communities. These results are not specific to traps, but could be applied for the use of tsetse control targets or other disease control tools, which are new to target communities.

The salient implication of this study is that tsetse control programs should plan and budget for active community involvement into control operations at all stages of the programs. Our findings suggest that more attention could be placed on sharing tools with, and passing on responsibilities to, the communities affected by HAT. Sensitisation activities would be best carried out before tsetse baits are deployed to give sufficient time for the community to absorb and distribute the message and information distributed should be carefully tailored to the local context. We expect that this bottom-up approach will significantly increase the level- and reduce the timeframe- of acceptance of control measures which will consequently improve efficacy and sustainability of control operations. This should especially be taken into consideration if tsetse control programs are implemented in areas new to such interventions.

It is possible that these implications for community participation would equally apply to the control of other neglected tropical diseases (NTDs). Evidence of the feasibility, cost-effectiveness and effective scaling up of control measures in community-driven interventions is mounting [43], [44]. However, community misunderstanding of disease control tools still poses a challenge to the success of such interventions. For example in Kenya, despite campaigns against schistosomiasis led by community volunteers, local suspicion of the purpose of treatment and surprise at the side effects of mass-drug administration persisted [45].

The obvious potential of community-participation in NTD control requires a paradigm shift from one-directional heath education towards equal dialogue with communities. Important pre-requisites for community participation are mutual understanding of the objectives of interventions as well as accessible, culturally acceptable, and effective vector control tools for prevention and drugs for timely treatment. We hope that this study will inspire similar research in other countries and other NTDs. Ultimately we hope these findings will contribute to the next generation of NTDs-, specifically vector-born disease- control strategies.

### Limitations

We are aware that this study has been conducted in collaboration with specific communities and that their views are determined by the local culture. However similar observations from other sites suggest that some results from this study are applicable in the wider context. Some communities thought we were representatives of HAT control programmes, and so some bias in overestimated perceived risk of HAT is expected. We managed this bias by communicating objective of this study and importance of capturing community views regardless of other activities linked to tsetse control trial.

## Supporting Information

Text S1 **Focus groups discussion topic guide.** Below is a list of topics and questions used to prompt discussion during the focus groups discussions Questions under each topic are numbered and probes used to stimulate discussion are indicated under bullet points.
(RTF)

Text S2 **Consent form.** The sample of this consent form was used to collect written or thumb-printed consent from each participant. This consent form was translated to Lugbara (local language of participants) and maintained under the same formatting as English version. Upon agreement with the statement, participants ticked the box on the right side of the page and signed or finger printed the line at the bottom. Researcher taking consent and literate witness when requested also signed the form. One (Lugbara copy) was left with the participant, while English copy was kept and stored in the project research station in Arua.
(RTF)
