# Supplementary material for: Community Acceptance of Tsetse Control Baits: A Qualitative Study in Arua District, North West Uganda
Source: PLoS Negl Trop Dis. 2013 Dec 12;7(12):e2579. doi: 10.1371/journal.pntd.0002579 (PMC3861179; doi:10.1371/journal.pntd.0002579)
Supplement: Text S1 — Focus groups discussion topic guide. Below is a list of topics and questions used to prompt discussion during the focus groups discussions Questions under each topic are numbered and probes used to stimulate discussion are indicated under bullet points. (RTF) [file pntd.0002579.s001.rtf]

Text S1: Focus Groups Discussion Topic Guide

Topic	Questions	
HAT burden	1. How much is HAT a burden?
·	Which five diseases are the most common in your community (list diseases raised by the group on board) 
·	Do you know HAT? Where does HAT lie within them?
·	Does it have worse effects compared to other diseases
2. How many HAT cases can you think off in your community in duration of your lifetime (ask to shout out the numbers)
3. How many of you had cases of HAT in your families/households (ask to lift hands)
4. How does HAT affect individual/family/community? (ask to think of particular case they know)
5. What are the community attitudes towards HAT cases?	
Perceived risk of contracting HAT	1. Do you know how does somebody contract HAT?
·	How?
2. Are you concerned about contracting HAT on a daily basis?
·	Why? 
·	Who is mostly under risk?
3. List examples mentioned by the group on board
4. Do you know tsetse flies?
·	Can you describe them?
5. Where do you mostly get bitten by tsetse flies?
6. Do you use anything to prevent being bitten?
7. Do you know if tsetse transmit any diseases in your community?
·	Which ones?	
Perception of tsetse traps and control strategies	1. Have you seen this object before (show tsetse trap)
·	Where have you seen it?
·	When have you seen it?
·	What did you think it was?
·	What was your reaction when you first saw this object? (explore further if any meanings are mentioned)
2. Explain the purpose of the traps 
·	What do you think about traps?
·	What do you like bout them?
·	Why? What is it you do like?
·	What don't you like about them?
·	Why? What is it you don't like?
·	List raised points on board
(Provide verbal summary of the main points raised by discussion)	
Recommendations	1. What could be done to reduce burden of HAT in your community?
2. How could you be involved in tsetse control?
·	Would you want to be involved?
·	why?
·	How?
CLOSING REMARKS: thank you/any questions, clarification/refreshments	
