# Supplementary material for: Community Acceptance of Tsetse Control Baits: A Qualitative Study in Arua District, North West Uganda
Source: PLoS Negl Trop Dis. 2013 Dec 12;7(12):e2579. doi: 10.1371/journal.pntd.0002579 (PMC3861179; doi:10.1371/journal.pntd.0002579)
Supplement: Text S2 — Consent form. The sample of this consent form was used to collect written or thumb-printed consent from each participant. This consent form was translated to Lugbara (local language of participants) and maintained under the same formatting as English version. Upon agreement with the statement, participants ticked the box on the right side of the page and signed or finger printed the line at the bottom. Researcher taking consent and literate witness when requested also signed the form. One (Lugbara copy) was left with the participant, while English copy was kept and stored in the project research station in Arua. (RTF) [file pntd.0002579.s002.rtf]

Text S2: Consent form

Participant Identification Number for this Study:_________

		                              
			 
1.	I confirm I have read and understood the information sheet dated......... (Version.......) for the above study.  I have had the opportunity to consider the information, ask questions and have had these answered satisfactorily.
2.	I understand that participation in this study is voluntary and I am free to withdraw   consent at any time, without giving a reason, without any penalties.
3.	I understand that data collected during the study, may be looked at by individuals from LSTM and from regulatory authorities.  I give permission for these individuals to have access to my records.

4.	I hereby declare that I have not been subjected to any form of coercion in giving this consent

5.	I agree / do NOT agree for any visual materials with my image (photos, video-clips) collected during this study to being stored and used for public disclosure (written publications, conferences, raising awareness meetings)                       
6.	I agree to take part in this study.
Signing this declaration does not affect your right to decline to take part in any future study.

			                  		     ____        			  __                  
Name of participant			Date			Signature
or

__________________________            _____________            _________________
Name of illiterate participant                    Date                              Signature 
and

__________________________           ____________                _________________
Name of literate witness                         Date                                  Signature

			                         ____	    ___            	______________       
Name of person taking 		          Date			Signature
Consent	

When complete: 1 copy for participant; 1 copy (original) for research file.
